# Optimizing short-term antibiotic treatment in patients with acute cholangitis: study protocol for an open-label randomized controlled trial (the BOLT-P3 trial)

**DOI:** 10.1186/s13063-025-09077-1

**Published:** 2025-09-01

**Authors:** Sakue Masuda, Yoshinori Imamura, Karen Kimura, Makomo Makazu, Jun Kubota, Hiroshi Takihara, Ryuhei Jinushi, Tomoaki Fujikawa, Kyohei Maejima, Aya Kawanishi, Ryuichi Yamamoto, Shogo Noda, Taiji Koyama, Ayumu Sugitani, Shomei Ryozawa, Kazuya Koizumi

**Affiliations:** 1 Department of Gastroenterology Medicine Center, Shonan Kamakura General Hospital, Kamakura, 247-8533 Japan; 2 Cancer Care Promotion Center, University of Fukui Hospital, Eiheiji, Fukui 910-1193 Japan; 3 Department of Gastroenterology, Uji Tokushukai Hospital, 145 Ishibashi, Makishima-Cho, Uji, Kyoto 611-0041 Japan; 4 Division of Gastroenterology, Saitama Medical University International Medical Center, 1397-1 , Yamane, Hidaka, Saitama Japan; 5 Center for Liver, Biliary Tract, and Pancreatic Diseases, Shonan Fujisawa Tokushukai Hospital, 1-5-1 Tsujido Kandai, Fujisawa, Kanagawa 251-0041 Japan; 6 Department of Gastroenterology, Chiba Nishi General Hospital, 107-1 Kanegasaku, Matsudo, Chiba 270-2251 Japan; 7 Division of Gastroenterology, Tokai University School of Medicine, 143 Shimokasuya, Isehara, Kanagawa 259-1193 Japan; 8 Department of Gastroenterology, Tokyo Nishi Tokushukai Hospital, 3-1-1 Matsubara-Cho, Akishima, Tokyo 196-0003 Japan; 9 Department of Gastroenterology, Fukuoka Tokushukai Hospital, 4-5 , Sugukita, Kasuga, Fukuoka Japan; 10 Division of Medical Oncology/Hematology, Department of Medicine, Kobe University Graduate School of Medicine, Kobe, 650-0017 Japan; 11 Department of the Institute of Biomedical Research, Sapporo Higashi Tokushukai Hospital, Hokkaido, 065-0033 Japan

**Keywords:** Antibiotic stewardship, Cholangitis, Endoscopic retrograde cholangiopancreatography, Antibiotic resistance, Duration of antibiotic therapy, Randomized control trial

## Abstract

**Background:**

Acute cholangitis (AC) frequently presents as a community-acquired infection and is associated with a high prevalence of antibiotic use among infectious diseases. The Tokyo Guidelines 2018 (TG18) recommend 4–7 days of antibiotic administration after biliary drainage. However, this recommendation lacks strong evidence of its effectiveness and is primarily based on heterogeneous clinical findings and expert opinions. Recent retrospective studies have advocated a shorter 1- to 3-day antibiotic course as effective for AC treatment, prompting the need to reassess the treatment duration to achieve therapeutic efficacy while minimizing resistance and adverse effects.

**Methods:**

We designed a multicenter, non-blinded, randomized trial to evaluate the efficacy of short-course therapy compared to standard-course therapy for AC management. The short-course therapy group will receive 1–3 days of intravenous (IV) antibiotic treatment after successful biliary drainage compared to 4–7 days of IV antibiotics after successful biliary drainage for the standard-course therapy group. The primary outcome is the clinical cure rate within 14 days from the endoscopic retrograde cholangiopancreatography (ERCP) procedure. Participants will be allocated to either treatment course using a minimization method in a non-blinded, randomized manner, with stratification factors including condition severity and facility. We determined that 210 participants would be required to achieve a statistical power of 90% with a one-sided significance threshold of 2.5% and a non-inferiority limit of 10%.

**Discussion:**

This phase 3 trial aims to determine the non-inferiority of short-course therapy over standard-course therapy. Shortening the duration of antibiotic administration may mitigate the emergence of resistant bacteria, adverse events, and reduce hospital stay length and healthcare costs.

https://jrct.niph.go.jp/re/reports/detail/73862

**Trial registration:**

This study was registered at the Japan Registry of Clinical Trials under registry number jRCT1031230709. Registered on 14 March 2024, https://jrct.niph.go.jp/re/reports/detail/73862

**Supplementary Information:**

The online version contains supplementary material available at 10.1186/s13063-025-09077-1.

## Background

The risk of developing resistant organisms increases with each additional day of antimicrobial therapy (1). Furthermore, prolonged antibiotic use has been reported to increase healthcare costs; escalate adverse events associated with antimicrobial therapy—such as *Clostridioides difficile* enteritis, invasive candidiasis, antibiotic-induced organ damage; and increase mortality rates in certain infections, including intra-abdominal infections (1–7). Therefore, optimizing antibiotic use in acute cholangitis (AC), which ranks as the second or fourth most prevalent cause of community-acquired bacteremia (8,9), is crucial for suppressing resistant bacteria and reducing antibiotic-associated adverse events (10).

The Tokyo Guidelines 2018 (TG18), a well-recognized set of guidelines for AC management, recommend antibiotic therapy and biliary drainage as the primary treatment modalities. TG18 suggests a standard duration of 4–7 days for antibiotic administration post-biliary drainage (11). However, it is noted that a duration of ≤ 3 days of antimicrobial therapy may suppress the development of resistant organisms (12).

A recent randomized controlled trial (RCT) compared 4- and 8-day antibiotic treatment durations and confirmed the non-inferiority of the shorter regimen (13). Additionally, recent retrospective studies have suggested that an even shorter course of antibiotics, lasting 1–3 days post-biliary drainage, may be effective in treating AC (14–16). These studies indicate that reducing the duration of antibiotic therapy does not compromise the clinical cure rate and may mitigate the risks associated with prolonged antibiotic use, including the development of resistant organisms and other adverse effects.

Despite the growing body of evidence, recommendations are predominantly based on small retrospective studies. Therefore, obtaining more robust evidence to refine and validate the optimal antibiotic therapy duration for AC is imperative to ensure maximal therapeutic benefit while minimizing adverse effects. This study aims to evaluate whether short-course therapy is non-inferior to standard-course therapy in terms of clinical cure rate. The research is crucial for developing evidence-based guidelines that balance the efficacy of treating AC with the need to prevent the emergence of resistant bacteria and reduce antibiotic-related adverse events.

## Methods

### Design

**Fig. 1:**
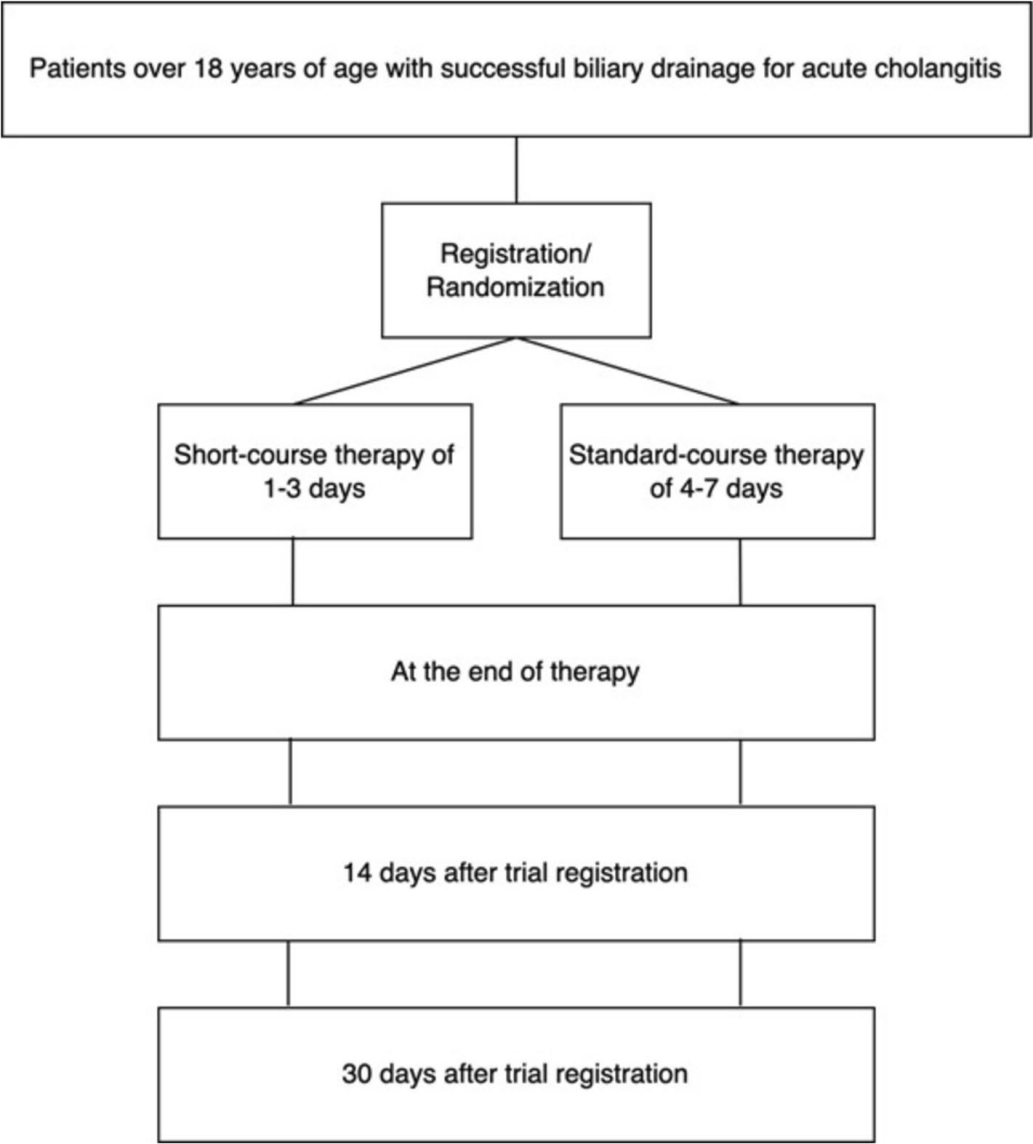
Study methodology

We aim to conduct a multicenter, open-label, randomized trial to assess the non-inferiority of short-course therapy compared with standard-course therapy for AC management after successful biliary drainage. A comprehensive report of the trial will be prepared in accordance with the Consolidated Standards of Reporting Trials (CONSORT) guidelines, including specific recommendations for non-inferiority studies (17). This study was registered with the jRCT under the identifier jRCT1031230709. The protocol of this study was developed according to the Standard Protocol Items Recommendations for Interventional Trials (SPIRIT) guidelines, with the SPIRIT checklist included as Additional file. A detailed diagram of the methodology used in this study is shown in Fig. 1.

### Setting

The study will be conducted at eight secondary or tertiary referral hospitals in Japan (Table 1).

**Table 1:** Participating institutions and investigators

Institutions	Investigators
Shonan Kamakura General Hospital	Sakue Masuda, MD
Uji Tokushukai Hospital	Hiroshi Takihara, MD
Saitama Medical University International Medical Center	Ryuhei Jinushi, MD
Shonan Fujisawa Tokushukai Hospital	Tomoaki Fujikawa, MD
Chiba Nishi Tokushukai Hospital	Kyohei Maejima, MD
Tokai University School of Medicine	Aya Kawanishi, MD
Tokyo Nishi Tokushukai Hospital	Ryuichi Yamamoto, MD
Fukuoka Tokushukai Hospital	Shogo Noda, MD

### Research implementation period

The study will be conducted from the date of the facility director’s approval until March 31, 2027, and includes the following phases: an enrollment period until March 31, 2026; a follow-up period until April 30, 2026; and a final phase for manuscript writing, academic presentations, and reporting from April 30, 2026, to March 31, 2027.

### Patients

Patients who meet the diagnostic criteria for confirmed or suspected AC according to the TG18 will be considered eligible for trial participation (18). Patients will be enrolled in the study when both the attending physician and the patient consent to participate in the trial, and the participant fulfills all eligibility criteria without meeting any exclusion criteria.

### Inclusion criteria

Participants must meet all of the following criteria:

1. Disease: Patients diagnosed with 140 AC according to the TG18, regardless of its benign or malignant nature. In patients with AC due to distal bile duct stent dysfunction, inclusion will be limited to those having the stent in place for > 30 days. We will exclude patients whose stent occlusion occur within 30 days of placement, as this may reflect technical or procedural issues unrelated to the antibiotic regimen. A similar exclusion criterion was used in a comparable randomized controlled trial [Short-course Antibiotics vs Standard Course Antibiotics in Patients With Cholangitis (COBRA), ClinicalTrials.gov ID: NCT05750966].
2. Age: ≥ 18 years.
3. Previous Treatment: Cases where biliary obstruction was successfully relieved through biliary drainage with endoscopic retrograde cholangiopancreatography (ERCP) within 48 h of hospitalization (13,19).
4. Consent: Written informed consent to participate in the trial was obtained from the patients or their legal representatives.

### Exclusion criteria

Participants will be excluded if they meet any of the following criteria:

1. Circulatory insufficiency with catecholamines at the time of ERCP.
2. Requiring intensive care unit (ICU) admission.
3. Hypothermia (35).
4. Recurrent cholangitis within three months.
5. Malignant or benign biliary stricture being equivalent to or beyond the Bismuth type II in the hepatic portal area or having an indeterminate cause of biliary obstruction (20).
6. Having anatomical changes due to surgery (e.g., biliary jejunal anastomosis excluding Billroth I).
7. Being diagnosed with concomitant pancreatitis based on the International Pancreatic Society/American Pancreatic Association guidelines (meeting two of the following criteria) (21): upper abdominal pain, serum amylase or lipase >3x upper limit of normal (ULN), and imaging signs of acute pancreatitis.
8. Concurrent cholecystitis according to the TG18 criteria (meeting one item from A and one from B and C):
9. Concurrent hepatic abscess.
10. Concurrent other infections.
11. Receiving continuous antibiotic administration for more than one week before registration.
12. Having complications from ERCP before registration (perforation; pancreatitis; bleeding; cholecystitis; or cardiopulmonary diseases due to sedation, e.g., aspiration, pneumonia, choking, and cardiac arrest, among others (26–28)).
13. Specific immunosuppressed states:
14. Being pregnant.
15. Being excluded from participation in the trial by the attending physician for other reasons.

### Ethics and informed consent

This trial will adhere to the ethical standards outlined in the Declaration of Helsinki and the guidelines for medical and health research involving human participants issued by Japan’s Ministry of Health, Labour and Welfare and Ministry of Education, Culture, Sports, Science and Technology (29). The ethical review boards of all involved hospitals approved the study protocol. Written informed consent was obtained from all participating patients and their legal proxies.

### Randomization and allocation concealment

Participants in the study will be assigned to one of the treatment groups in a balanced 1:1 ratio within 24 h of the successful completion of biliary drainage. The allocation process will utilize a stochastic minimization method centrally managed by the study’s primary center at the Department of Gastroenterology, Shonan Kamakura General Hospital. A specialized electronic data capture system will be used for randomization and data collection. Stratification factors will include condition severity and facility as criteria.

### Trial interventions

This study will compare short-course (1–3 days) and traditional standard-course (4–7 days) antibiotic therapy for AC management after successful biliary drainage.

The antibiotics administered to study participants are commercially available and approved for use in Japan. They will be administered at the standard dosage specified in the package insert. The initial antibiotics will be selected at the discretion of the treating physician. The selected antibiotics can be changed to other antibiotics during treatment based on culture/susceptibility test results or any suspected or occurring adverse reactions in the patient. Adjustments based on patient renal function or other criteria will be performed when judged appropriate and necessary by the attending physician.

### Extension of the antibiotic administration period

Extending the treatment period in AC is not mandatory, even with positive blood culture results. Several retrospective studies have shown that short-term administration of antibiotics (2–3 days) does not worsen outcomes, even in cases with positive blood cultures (16,30). In response, the Société de Pathologie Infectieuse de Langue Française (SPILF) recommends a short-term antibiotic administration period (within 3 days), even for cholangitis with bacteremia (31). However, an extension of the antibiotic administration period is permissible and recommended at the discretion of the attending physician in cases that meet any of the following criteria:

1. Antibiotics were administered for over 72 h during the short-course therapy until the blood culture sensitivity results were known.
2. For blood cultures positive for *Staphylococcus*, antibiotic therapy for more than 14 days is recommended, followed by a confirmatory negative blood culture (32).
3. Recommending a change of antibiotics if the blood culture sensitivity results show that the antibiotic spectrum is inappropriate to cover the infection. The duration of treatment after changing antibiotics will be 24–72 h for the short-course therapy group and 96–168 h for the standard-course therapy group.
4. Allowing a change or extension of antibiotics with a lack of improvement in clinical symptoms (chills, body temperature > 37.5 °C on the third day of enrollment (33), abdominal pain, or jaundice). However, antibiotic administration should be limited to a maximum of 7 days.
5. Even if antibiotics are discontinued once, it is permissible to restart them in cases of poor drainage (if the T-bilirubin level on the third day after ERCP abnormally exceeds 80% of the level before ERCP (34)). However, antibiotic administration should be limited to a maximum of 7 days.
6. Extending the antibiotic administration period up to 14 days, including oral administration, is permissible at the discretion of the attending physician in patients already diagnosed with artificial valve replacement surgery, previous infectious endocarditis, or congenital heart disease (single ventricle, complete transposition of the great vessels, tetralogy of Fallot) or if gram-positive coccal bacteremia is confirmed.
7. Extending the antibiotic administration period according to concurrent infection is recommended if other infections, such as liver abscess, cholecystitis, or intra-abdominal abscess, are discovered after starting the trial. Cholecystitis and intra-abdominal abscesses should be treated for 1–4 days after appropriate source control (35–37). Evidence regarding liver abscess treatment is scarce; nevertheless, the duration of antibiotic treatment should be tailored to the patient’s condition, ranging from 8 to 69 days (38). Recommending an appropriate antibiotic administration period in cases where the source of infection cannot be controlled is difficult; nevertheless, the general recommendation is 1–2 weeks for cholecystitis and 2–6 weeks for liver abscesses.

### Assessment and follow-up

**Fig. 2:**
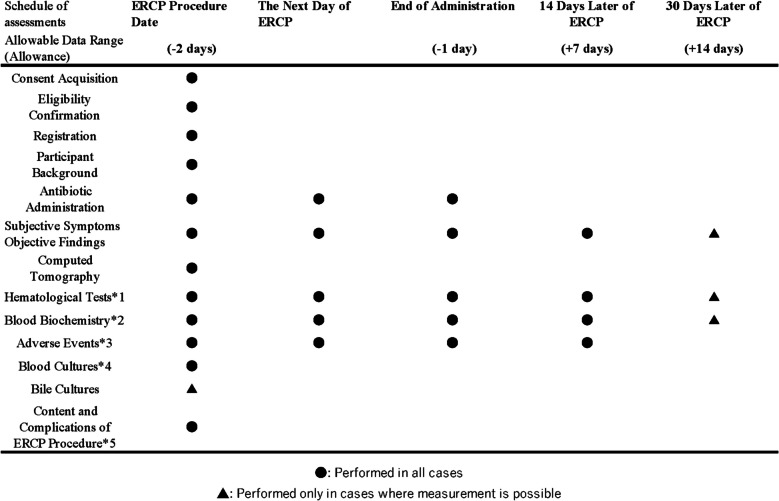
Schedule of assessments. *1: Hematological tests, including white blood cells, white blood cell differential, red blood cells, hemoglobin, hematocrit value, and platelets. *2: Blood biochemistry tests, including creatinine, total bilirubin, direct bilirubin, aspartate aminotransferase, alanine aminotransferase, alkaline phosphatase, gamma-glutamyl transferase, C-reactive protein, and amylase. *3: Adverse events, including rash, diarrhea, *Clostridioides difficile* enteritis, liver damage, and kidney damage. *4: In this trial, blood cultures were collected within 24 h of initial contact with the treating physician before the start of antibiotic administration. *5: Endoscopic retrograde cholangiopancreatography (ERCP) procedure details (stone removal, plastic stent, metallic stent, and balloon dilation, among others) and adverse events (bleeding, perforation, pancreatitis, bile leak, cholangitis, or liver abscess)

The schedule of assessments is shown in Fig. 2. Clinical assessments will be performed on the date of the ERCP procedure, the day after ERCP, at the end of antibiotic administration, and on days 14 and 30 after ERCP.

In this trial, direct follow-up by the principal investigator and sub-investigators is the primary approach. For participants who miss follow-up visits, efforts will be made to collect data via telephone contact whenever possible.

### Outcome measures

The primary outcome was defined as the rate of cases that achieved clinical cure within 14 days and survived without recurrence, according to the guidelines recommended by the U.S. Food and Drug Administration for complicated urinary tract infection outcomes (39). Clinical cure refers to the resolution of all pre-treatment clinical symptoms (chills, fever > 37.5 °C, abdominal pain, jaundice, impaired consciousness). Recurrence was defined as the reappearance of clinical signs suggestive of cholangitis in a case that had completed antibiotic therapy and achieved clinical cure, necessitating the resumption of antibiotic administration. Clinical signs suggestive of cholangitis recurrence include any of the following: (1) reappearance of clinical signs of cholangitis; (2) manifestation of signs of infection in the liver, pancreas, or biliary tract; or (3) other signs of infection considered related to the initial cholangitis episode (30,40).

The secondary outcomes include the following:

1. Recurrence rate within 30 days
2. Mortality rate within 30 days
3. Total number of days of antibiotics required for both groups within 30 days
4. Total number of days of hospitalization required for both groups within 30 days
5. Clinical cure rates by severity
6. Clinical cure rates by the presence or absence of fever at the end of antibiotic treatment
7. Clinical cure rates in older populations aged ≥ 75 years (18,41)
8. Clinical cure rates by blood culture results
9. Clinical cure rates by bile culture results
10. Clinical cure rates based on antibiotic susceptibility before ERCP
11. Clinical cure rates based on antibiotic administration duration before ERCP
12. Clinical cure rates based on the time from admission to the ERCP procedure
13. Clinical cure rates based on AC etiology
14. Treatment costs
15. Incidence of the following adverse events within 14 days:
  (a). Rash
  (b). Diarrhea (defined as ≥ 3 loose stools per day (42))
  (c). *C. difficile* enteritis (42), confirmed experimentally and through clinical signs

Clinical signs: Presence of diarrhea (≥ 3 formless stools within 24 h).

Experimental confirmation:

- Detection of *C. difficile* toxin A and/or toxin B in stool
- Positive *C. difficile* stool antigen
- Positive *C. difficile* culture
- Detection of specific genes of *C. difficile* by molecular diagnostic methods such as polymerase chain reaction
- Detection of *C. difficile toxins *by the cell cytotoxicity neutralization assay
  - Drug-induced liver injury (43), according to any of the following criteria:
- Hepatic dysfunction (alanine aminotransferase [ALT] elevation exceeding 5 × ULN)
- Alkaline phosphatase (ALP) elevation exceeding 2 × ULN
- ALT elevation exceeding 3 × ULN, and total bilirubin concentration exceeding 2 × ULN

Additionally, drug-induced liver injury will be diagnosed if there is no observed worsening of biliary duct dilation upon re-evaluation with imaging diagnostics, or if there is a re-elevation of hepatic and biliary enzymes following an initial decrease.

- Acute kidney injury (44), according to any of the following criteria:
  - An increase in serum creatinine by ≥ 0.3 mg/dL (≥ 26.5 µmol/L) within 48 h from the time of initial administration of antibiotics at the start of the trial (or from the time they were first used, if before the start of the trial)
  - An increase in serum creatinine to ≥ 1.5 times the baseline from the time of initial administration of antibiotics at the start of the trial until the end of administration

Serum creatinine results obtained by the attending physician within this period will be included in the evaluation, even if measured on non-scheduled test days.

The baseline during normal times was defined as the creatinine value within 1 year before the start of the trial. In cases with an unknown baseline, the lowest value within 7 days before the end of antibiotic administration will be used as the baseline.

Adverse events will be graded using the Common Terminology Criteria for Adverse Events version 5.0 (45).

### Sample size

The clinical cure rates for short- and standard-course therapies were estimated using our retrospective dataset. According to a dataset of 361 patients that closely matched the control group in this trial, the clinical cure rate was 95.1% (135/142) in the short-course therapy group and 95.0% (208/219) in the standard-course therapy group, with an overall rate of 95.0% (343/361). This is consistent with the results reported in the literature (46,47). Therefore, a clinical cure rate of 95% was used in both groups for sample size calculation in this trial. The number of patients required for analysis was determined to be 200, with 100 cases per group, assuming a one-sided significance level of 2.5%, power of 90%, and a non-inferiority margin of 10% (39). Anticipating loss to follow-up and other contingencies, the target enrollment was set at 210, with 105 cases per group.

### Statistical analysis

Regarding the primary endpoint, the clinical cure rate with 95% confidence intervals will be compared between the short-course therapy group (24–72 h) and the standard-course therapy group (96–168 h) in the full analysis set (FAS), which will include all registered participants who receive any part of the treatment protocol. Subgroup analyses will be conducted based on severity, age, AC etiology, and other baseline factors using the confidence intervals of the differences and logistic regression models. Interaction effects will be examined. The primary analysis population is the FAS; however, analyses using the per-protocol set (PPS), which includes participants from the FAS who had no study protocol violations and were compliant with the protocol, will also be conducted to confirm that the same conclusions are drawn from both sets.

Secondary endpoints will also be examined in both the FAS and PPS sets; these will include the following variables within 30 days: the recurrence and mortality rates, required days of antibiotic use, required days of hospitalization, and treatment costs.

A safety analysis will be conducted among the participants who received the study treatment at least once. All adverse events will be reported, and serious adverse events (SAE) will be tallied separately. Adverse events observed after administration of the trial drug will be tabulated by group, and the number of occurrences, cases, and incidence rates will be calculated and compared between groups. All adverse events will be analyzed, regardless of causality. Descriptive statistics will be calculated by group for clinical laboratory tests, and clinically significant differences will be highlighted. Other parameters will be analyzed by variable type; descriptive statistics will be calculated for continuous variables and frequencies tallied for categorical variables in each group.

Continuous variables will be reported as medians with interquartile ranges, and categorical variables will be reported as numbers with percentages. Continuous and categorical variables will be compared using the Mann–Whitney *U* and chi-square tests, respectively. Risk differences with 95% confidence intervals (CIs) will be calculated for binary outcomes, and the two-sided significance level for all tests will be set at *p* < 0.05. All statistical analyses will be performed using EZR (Saitama Medical Center, Jichi Medical University, Saitama, Japan), a graphical user interface for R version 4.2.3 (R Foundation for Statistical Computing, Vienna, Austria), which is a modified version of the R commander designed to include additional biostatistical functions (48).

### Trial oversight

This study will be conducted at the Center for Gastroenterology of the Shonan Kamakura General Hospital. The data center is co-located with data management personnel to facilitate overseeing centralized data monitoring throughout the trial period. The Operations Committee played a role in formulating the protocol and will supervise the progression of the trial (Table 2). No patients or members of the public were involved in the design of this study.

**Table 2:** Steering Committee

Role in this study	Name	Institution
Principal Investigator	Sakue Masuda	Department of Gastroenterology Medicine Center, Shonan Kamakura General Hospital
Supervision/Overall Guidance	Yoshinori Imamura	Division of Medical Oncology/Hematology, Department of Medicine, Kobe University Graduate School of Medicine
Research Support	Norimasa Harada	Clinical Research Support Office, Shonan Kamakura General Hospital
Research Collaborator	Keiko Aso	Clinical Trial Center, Shonan Kamakura General Hospital
Data Management	Karen Kimura	Department of Gastroenterology Medicine Center, Shonan Kamakura General Hospital
Event Adjudication Committee	Kento Shionoya	Department of Gastroenterology and Hepatology, Tokyo Medical University
Event Adjudication Committee	Haruki Uojima	Genomic Medicine Research Project, National Center for Global Health and Medicine
Event Adjudication Committee	Hidaka Hisashi	Department of Gastroenterology and Hepatology, Kitasato University School of Medicine
Study Statistician	Ayumu Sugitani	Department of the Institute of Biomedical Research, Sapporo Higashi Tokushukai Hospital
Study Secretariat		Shonan Kamakura General Hospital
Project Management		Shonan Kamakura General Hospital

The principal investigator reviews cumulative enrollment each month and sends a progress report via email to all collaborating sites, encouraging additional recruitment as needed. An investigator kick-off meeting was held at study launch, and the principal investigator remains in close contact with participating sites by email or teleconference so that even minor operational issues can be resolved promptly.

Because all antibiotic therapy is delivered by intravenous infusion under the direct supervision of healthcare professionals, patient-side adherence measures (e.g., tablet counts) are not applicable. At the initiation meeting, all sites were instructed to follow the protocol strictly.

At the coordinating center, both a Clinical Research Center and a Clinical Trial Center have been established. Clinical Research Centers are also present at each participating site. The Clinical Research Centers are responsible for protocol development and amendments, and coordination with participating sites. The Clinical Trial Center provides operational advice and liaises with monitoring and auditing bodies. Several full meetings were held at the start of the study. Although regular full meetings are not planned to be held after that point, close consultations are conducted with the principal investigator whenever needed to support decision-making and trial management—even on minor matters.

In case of any protocol amendments, the principal investigator will notify all participating centers of the changes. A copy of the revised protocol will be filed in the Investigator Site File at each site. Any protocol deviations will be documented using a breach report form. The clinical trial registry entry (e.g., jRCT) will be updated accordingly.

### Monitoring and audit

In this trial, monitoring will be conducted by Mirai Iryo Research, Inc. and the monitoring staff of the lead facility. Biannual routine monitoring sessions will be performed based on the monitoring reports prepared by the Research Administration Office. These sessions involve a comprehensive meeting with all or some representatives of group leaders, principal researchers, administrative offices, the Efficacy and Safety Evaluation Committee, and participating institutions. Interim analysis was not planned for this study because the follow-up period for the primary analysis is short, and any safety issues with the participants could be ascertained through periodic monitoring. Routine on-site monitoring will occur at least once every 3 months to review study progress and protocol compliance, with additional visits triggered by SAEs, major protocol deviations, or other emerging issues. Key source-data verification will include informed consent, eligibility, primary and secondary endpoints, SAEs, and protocol compliance. Adherence—including the scheduled start time, infusion rate, and duration of each dose—will be verified during these visits and during independent audits (Supplementary data 1, 2, and 3). A risk-based sampling strategy will be applied, with 100% verification of informed consent and SAEs and random sampling for efficacy endpoints.

The Clinical Research Support Unit of the Japanese Society for Regenerative Medicine (CRS JSRM) will provide education and conduct audits aimed at improving the scientific and ethical quality of research. Auditors will visit the representative facilities of the trial to verify the facility’s Institutional Review Board (IRB) documents and patient consent forms and reconcile the data on forms submitted to the office with medical records, following the audit plan developed by the CRS JSRM Unit. Auditing is scheduled to take place three times: October 24, 2024; June 17, 2025; and April 2026. On-site, document, and operational audits will be conducted to ensure data integrity, compliance with Standard Operating Procedures, and proper trial management. The audit is independent of the sponsor and investigators.

### Confidentiality

A designated party is responsible for storing all study-related information and materials; this responsibility includes the retention of records relevant to the study (copies of application documents, notification documents from the director, various application forms and reports, study-related materials, and other documents or records necessary to ensure data reliability). Furthermore, personal information will be protected during the disposal of documents or materials by shredding and incinerating physical documents (for electronic data, this involves using dedicated data deletion software or physical destruction to render the data irrecoverable). The principal investigator will take measures to ensure that these documents are not accidentally or prematurely destroyed.

Retention period: Documents will be retained for 10 years after the study’s end is reported or for 8 years after the final results are published, whichever period is longer.

## Discussion

This trial assesses whether short-course antibiotic therapy of 24–72 h for AC with appropriate biliary drainage is non-inferior to standard-course therapy of 96–168 h. A retrospective cohort study indicated comparable effectiveness between short-course and standard-course therapy for AC, with complications appearing to be more frequent with standard-course therapy (14–16,49). Should short-course therapy prove to be non-inferior, it may offer several advantages over standard-course therapy, including shorter hospital stays, reduced potential side effects from antibiotic treatment, lower costs, and a decreased likelihood of developing antibiotic resistance (1–6). However, if the non-inferiority of the trial treatment is not demonstrated, then the current standard treatment duration will be considered necessary and sufficient for this population.

This study will focus on several specific conditions. The antibiotic therapy duration may not affect outcomes in severe cases of AC (13). However, in the ICU, diseases such as septic shock can result in a mortality rate of approximately 20% (50). Therefore, patients admitted to the ICU or in an equivalent critical state will be excluded from the study. Retrospective studies have suggested that positive blood cultures and persistent fever do not affect outcomes (16,30,46,47). Consequently, we decided not to alter the duration of antibiotic therapy in cases where fever persisted, except in those with a high risk of infective endocarditis or *Staphylococcus aureus* detection, to maintain the same duration in cases with positive blood cultures (32,51). The applicability of standard or short-term treatment for patients with malignant tumor-induced multiple hilar biliary obstructions remains unclear. However, retrospective studies have reported frequent drainage inadequacies requiring repeated biliary drainage procedures in cases with multiple hilar biliary obstructions (20,52,53). These cases will be excluded because their investigation would require an evaluation of drainage methods, which is beyond the scope of assessing antibiotic therapy duration. Additionally, conditions such as pyogenic liver abscesses and acute cholecystitis are often observed simultaneously, necessitating different antibiotic treatment strategies for cholangitis. Therefore, cases with these comorbidities identified before registration will be excluded, while for patients who develop these conditions post-registration, the antibiotic therapy duration will be adjusted as appropriate for each condition (35–38).

The proposed trial has several inherent limitations. First, designing a double-blind trial was not feasible owing to practical considerations. Nevertheless, we believe that the defined outcomes are clear and will not be significantly compromised by the trial’s open-label design, as demonstrated in a similar previous study (13,35). Second, clinical outcomes will be assessed by attending physicians, which introduces an inherent risk of subjectivity in outcome assessment. However, the clear definitions of trial outcomes and the high cure rate of AC are expected to minimize this issue. Third, patients with multiple hilar biliary obstructions classified as Bismuth II or higher based on computed tomography and ERCP findings are considered susceptible to poor drainage and adverse prognosis and will be excluded. Therefore, a separate prospective study for this group will be necessary in the future.

Similar randomized controlled trials (RCTs) were conducted in India (13) and the Netherlands (COBRA trial, NCT05750966). An RCT conducted in India demonstrated the non-inferiority of a shorter antibiotic therapy duration within the range recommended by the TG18. In contrast, the treatment group in the COBRA study was quite aggressive, with an antimicrobial administration period of only 1 day, which does not allow the reflection of blood culture results. This trial is distinct from these two RCTs as it aims to examine a short-course therapy duration of 1–3 days for AC in Japan. Japan’s setting differs from that of India—known for its extremely high prevalence of resistant bacteria—and the Netherlands—known for its low prevalence (16,30,54–58). Currently, RCTs are independently conducted in different countries, but insights from multiple and diverse environments are beginning to emerge. We believe that this trial will contribute to advancing antibiotic stewardship by establishing a novel and optimized duration of antibiotic therapy for AC, thereby reducing the adverse events associated with antibiotic use, including increased antibiotic resistance.

## Trial status

The protocol was initially on version 1.3 when patient recruitment began on June 1, 2024, and the first patient was recruited on June 5, 2024. During the recruitment phase, the protocol was updated to version 2.2, dated April 3, 2025. The trial is scheduled to end on March 31, 2027.

## Supplementary Information

Supplementary Material 1.

Supplementary Material 2.

Supplementary Material 3.

## Data Availability

The datasets used and/or analyzed in the current study are available from the corresponding author upon reasonable request.

## Abbreviations

AC: Acute cholangitis
TG18: Tokyo Guidelines 2018
IV: Intravenous
CONSORT: Consolidated Standards of Reporting Trials
SPIRIT: Standard Protocol Items Recommendations for Interventional Trials
ERCP: Endoscopic retrograde cholangiopancreatography
ICU: Intensive care unit
ULN: Upper limit of normal
CRP: C-reactive protein
WBC: White blood cell
HIV: Human immunodeficiency virus
SPILF: Société de Pathologie Infectieuse de Langue Française
SAE: Serious adverse events
RCT: Randomized control trial
NEWS: National Early Warning Score
ITT: Intention to treat
*C. difficile*: *Clostridioides difficile*
ALT: Alanine aminotransferase
ALP: Alkaline phosphatase
FAS: Full analysis set
PPS: Per protocol set
CRS JSRM: Clinical Research Support Unit of the Japanese Society for Regenerative Medicine
IRB: Institutional Review Board
