# Supplementary material for: Optimizing short-term antibiotic treatment in patients with acute cholangitis: study protocol for an open-label randomized controlled trial (the BOLT-P3 trial)
Source: Trials. 2025 Sep 1;26:324. doi: 10.1186/s13063-025-09077-1 (PMC12400583; doi:10.1186/s13063-025-09077-1)
Supplement: Supplementary file 1 — Supplementary Material 1. [file 13063_2025_9077_MOESM1_ESM.docx]

# Audit Plan (Individual Audit)

Date: June 12, 2025

To: Ms. Sakue Masuda

Version: 1

Auditor: Naomi Miyazawa

## Audit Type

☑ On-site Audit ☑ Document Audit ☑ Operational Audit

## Type of Audit

☑ Routine Audit ☐ Follow-up Audit

## Study Title

Optimizing Short-term Antibiotic Treatment in Acute Cholangitis: rationale and study protocol for an open-label randomized controlled trial - The BOLT-P3 Trial (Biliary Optimal Limited Treatment - Phase 3)

## Study ID

jRCT1031230709 / UMIN000054071

## Audit Target Institution

Shonan Kamakura General Hospital, Gastroenterology Center
1370-1 Okamoto, Kamakura, Kanagawa, Japan

## Audit Details

This audit will be conducted from three perspectives—on-site, document, and operational audit—to confirm the proper and ethical conduct of the study. The progress of the study based on the protocol will be reviewed through inspection of source documents, data, and records, along with review of SOPs and site management.

In this audit, based on the current status of subject enrollment, the audit will also confirm items that were not reviewed in the previous audit, including the completeness of contract documentation, accuracy and consistency of data, and the overall conduct of the study.

## Audit Standards

Ethical Guidelines for Medical and Health Research Involving Human Subjects (Japan)

## Documents to be Reviewed

[On-site Audit] Protocol, source documents (data, records), SOP management, Site-level management (investigational drug, equipment)
[Document Audit] Protocol, SOPs, data records (integrity), monitoring reports, overall document control
[Operational Audit] Procedures, execution status (personnel, equipment, funding), ethics committee, monitoring, regulatory compliance, risk management, study progress (periodic and adverse event reporting)

## Scheduled Audit Date and Time

June 17, 2025, 10:00–16:00

## Remarks

This audit will focus on updates since the previous audit.

Original Plan Date: June 12, 2025

English Version Issued: August 8, 2025
