# Supplementary material for: Optimizing short-term antibiotic treatment in patients with acute cholangitis: study protocol for an open-label randomized controlled trial (the BOLT-P3 trial)
Source: Trials. 2025 Sep 1;26:324. doi: 10.1186/s13063-025-09077-1 (PMC12400583; doi:10.1186/s13063-025-09077-1)
Supplement: Supplementary file 2 — Supplementary Material 2. [file 13063_2025_9077_MOESM2_ESM.docx]

**Audit Plan (Overall)**

Date: June 12, 2025

To: Ms. Sakue Masuda

Version: 2

Auditor: Naomi Miyazawa

## Study Title

Optimizing Short-term Antibiotic Treatment in Acute Cholangitis: rationale and study protocol for an open-label randomized controlled trial - The BOLT-P3 Trial (Biliary Optimal Limited Treatment - Phase 3)

## Study ID

jRCT1031230709 / UMIN000054071

## Audit Plan and Schedule

This audit plan is developed in accordance with a risk-based approach. Audit activities are prioritized based on potential impact on subject safety, data integrity, and protocol compliance, ensuring efficient use of audit resources and focus on critical processes.

| Audit Type | Details | Planned Date(s) |
| --- | --- | --- |
| On-site Audit | Protocol, source documents (data, records), SOP management, Site-level management (investigational drug, equipment) | Oct 24, 2024 June 17, 2025 Apr 2026 |
| Document Audit | Protocol, SOPs, data records (integrity), monitoring reports, document control (including final study report in Apr 2026) | Oct 24, 2024 June 17, 2025 Apr 2026 |
| Operational Audit | Procedures, execution status (personnel, equipment, funding), ethics review committee, monitoring status, regulatory compliance, risk management, progress reporting (including periodic and adverse event reports) | Oct 24, 2024 June 17, 2025 Apr 2026 |

Original Plan Date: June 12, 2025

English Version Issued: August 8, 2025
