# Supplementary material for: Optimizing short-term antibiotic treatment in patients with acute cholangitis: study protocol for an open-label randomized controlled trial (the BOLT-P3 trial)
Source: Trials. 2025 Sep 1;26:324. doi: 10.1186/s13063-025-09077-1 (PMC12400583; doi:10.1186/s13063-025-09077-1)
Supplement: Supplementary file 3 — Supplementary Material 3. [file 13063_2025_9077_MOESM3_ESM.docx]

# Monitoring Plan

Version 3 – Date: February 27, 2025

This plan complements the separately defined Standard Operating Procedures for Monitoring in Clinical Research (SOP, Version 2), and outlines the monitoring strategy, specific procedures, and other essential matters to ensure appropriate monitoring conduct.

## Study Details

Study Title: A Multicenter, Open-label, Randomized Phase III Trial on Short-term Antimicrobial Therapy for Acute Cholangitis

EC/CRB Code: 2418

Study Site: Shonan Kamakura General Hospital, Tokushukai Medical Corporation

Primary Outcome: The proportion of patients who achieve clinical improvement without recurrence and remain alive within 14 days from the date of ERCP.

Study Period: From Institutional Review Board approval until March 31, 2027
 - Recruitment period: until March 31, 2026
 - Follow-up period: until April 30, 2026
 - Reporting and publication period: April 30, 2026 – March 31, 2027

Risk Level: Risk Level 3 (Refer to Appendix 1 of the SOP)

## Personnel

Principal Investigator: Dr. Sakue Masuda, Department of Gastroenterology

Co-Investigators: Dr. Kazuya Koizumi, Dr. Makomo Makazu, Dr. Karen Kimura (Department of Gastroenterology)

Hospital Liaison: Dr. Sakue Masuda, Department of Gastroenterology

Monitors: Clinical Trial Monitoring Division, Research Support Headquarters, Mirai Medical Research Center, Inc.

- Kumiko Cho
 - Miki Nakahara
 - Kaori Yoshimoto

## Monitoring

### Monitoring Items

■ Informed consent
■ Serious Adverse Events (SAEs)
■ Eligibility
■ Primary endpoint
■ Secondary endpoints
■ Compliance with the Clinical Trials Act, related guidelines, and study protocol
■ Others (e.g., confirmation of jRCT registration, tracking progress)

### Schedule / Frequency

Monitoring will be conducted at least once every 3 months to confirm the study’s progress.
In addition, monitoring may be conducted on an ad-hoc basis under the following conditions:
 - Upon reporting of a serious adverse event to the ethics committee
 - Upon reporting of a significant protocol deviation
 - At the end of the study
 - When any concerns arise during the conduct of the study

### Monitoring Methods

Monitoring will follow Article 7 of SOP (Version 2.0):
1. Informed consent: Confirm the original or copy of the signed consent form.
2. Eligibility: Confirm to the extent possible using source documents (e.g., medical records).
3. Primary endpoint: Compare case report forms (CRFs) with source documents such as medical records.
4. Secondary endpoints: Confirm CRF entries to the extent possible.
5. Safety (all adverse events): Confirm SAEs from SAE report forms.

### Sampling Strategy

1. SAEs and informed consent: 100% monitoring of all cases.
2. Eligibility: Review the first 3 enrolled cases.
3. Primary and secondary endpoints: Randomly sample 1 in every 10 cases.
4. Protocol or regulatory deviations: Check only for deviations related to monitored items.

### Reporting of Monitoring Results

Findings based on the monitoring records will be summarized in a monitoring report, including any important issues such as adverse events or protocol violations. The report will be submitted to the supervisor for approval.
After obtaining approval, the finalized monitoring report will be submitted to the Principal Investigator.
